# Gray space and default mode network-amygdala connectivity

**DOI:** 10.3389/fnhum.2023.1167786

**Published:** 2023-08-30

**Authors:** Julia C. Harris, Michael T. Liuzzi, Carlos Cardenas-Iniguez, Christine L. Larson, Krista M. Lisdahl

**Affiliations:** ^1^Department of Psychology, University of Wisconsin-Milwaukee, Milwaukee, WI, United States; ^2^Department of Population and Public Health Sciences, Keck School of Medicine, University of Southern California, Los Angeles, CA, United States

**Keywords:** fMRI, resting state, gray space, amygdala, default mode network

## Abstract

**Introduction:**

Aspects of the built environment relate to health factors and equity in living conditions, and may contribute to racial, ethnic, or economic health disparities. For example, urbanicity is linked with negative factors including exposure to gray space (e.g., impervious surfaces such as concrete, streets, or rooftops). While there is existing research on access to green space and urbanicity on some mental health and cognitive outcomes, there is limited research on the presence of *gray* space linked with cognitive functioning in youth. The goal of this study was to investigate the link between gray space and amygdala-default mode network (DMN) connectivity.

**Methods:**

This study used data from the ABCD Study. Participants (*n* = 10,144; age *M* = 119.11 months, female = 47.62%) underwent resting-state fMRI acquisition at baseline. Impervious surfaces (gray space) were measured via the Child Opportunity Index (COI). To examine the relationship between presence of gray space and -amygdala-DMN (left/right) connectivity, we employed linear mixed effects models. Correlations were run between amygdala-DMN connectivity and internalizing and externalizing symptoms. Finally, *post hoc* sensitivity analyses were run to assess the impact of race.

**Results:**

More gray space, adjusting for age, sex, and neighborhood-level variables, was significantly associated with increased left amygdala-DMN connectivity (*p* = 0.0001). This association remained significant after sensitivity analyses for race were completed (*p* = 0.01). No significant correlations were observed between amygdala-DMN and internalizing or externalizing symptoms.

**Discussion:**

Findings suggest gray space was linked with increased left amygdala-DMN connectivity, circuits that have been implicated in affective processing, emotion regulation, and psychopathology. Thus gray space may be related to alterations in connectivity that may enhance risk for emotion dysregulation. Future investigation of these relationships is needed, as neuroimaging findings may represent early dysregulation not yet observed in the behavioral analyses at this age (i.e., the present study did not find significant relationships with parent-reported behavioral outcomes). These findings can help to inform future public policy on improving lived and built environments.

## Introduction

**FIGURE 1:**
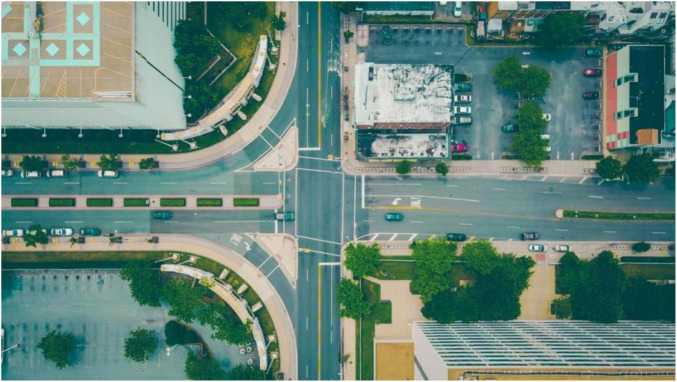
Example photo of gray space (e.g., roads, parking lots, rooftops) and green space (e.g., trees, grass). Photo by Tim Trad on Unsplash.

The built environment plays a crucial role in cognitive, emotional, and brain development. The built environment can be made up of many types of land including blue (e.g., sea and coastlines), brown (e.g., pervious surfaces without vegetation), gray (e.g., impervious surfaces), or green spaces (e.g., vegetation). Importantly, the rise of urbanization in the United States has led to reduced access to nature and an increased concentration of gray spaces. Gray space refers to the presence of impervious land including buildings, concrete, parking lots, roads, and rooftops (see Figure 1; Acevedo-Garcia et al., 2020). While urbanization has many benefits to society, it is associated with increased levels of chronic mental health outcomes (e.g., anxiety and depression; Wang, 2004; Peen et al., 2010; Lederbogen et al., 2011) and biological stress responses (e.g., hypertension) that lead to oxidative stress, inflammation, and neuronal injury (Bernabe-Ortiz et al., 2017; Xu et al., 2018), which in turn may impact brain development among youth. Studies have provided evidence that built environmental factors during childhood and adolescence impact brain function and structure. For example, Rakesh et al. (2022) found associations between different socioeconomic status variables (e.g., parent education, income, and the Area Deprivation Index, a composite encompassing housing quality, rankings of neighborhoods, etc.) and child brain structure (e.g., reduced cortical thickness of frontal, parietal, and occipital brain regions). While studies have investigated factors of the physical built environment such as air pollution, heat exposure, and green space on brain health and neurodevelopmental outcomes, research has not investigated gray space exposure.

While causal relationships between gray space, brain development, and mental health among youth are not well understood, it is posited that access to nature may be a buffer against the development of mental illness via a decrease in rumination by reducing the biological stress response (Bratman et al., 2012; Dadvand et al., 2012, Sudimac et al., 2022). Furthermore, increased access to green space can provide more social opportunities and lead to a reduction in cortisol and a decrease in blood pressure, which can in turn improve emotion regulation and other mental health concerns including anxiety, depression, and stress coping (Fan et al., 2011; Cohen-Cline et al., 2015). Inversely, a lack of access to nature and increased exposure to gray space may reduce opportunities for the restorative effects of the environment (e.g., stress reduction, social opportunities). However, limited research has been conducted on the effects of gray space as a land type on brain connectivity in regions implicated in psychopathology (e.g., affective processing). Understanding the role of gray space in development of brain circuits linked to psychopathology is necessary, as gray space is associated with less access to nature, and, in some cases, with other risk factors, such as extreme heat and increased air and noise pollution (Harlan et al., 2006; Hajat and Kosatky, 2010). Thus, future research investigating prospective and large-scale neuroimaging are needed to identify potential neurobiological mechanisms that may underlie the link between the built environmental, including gray space, and brain development while considering complex social drivers of health (e.g., Fan et al., 2021).

The amygdala is implicated in many aspects of functioning, including emotion regulation, reward and threat processing, and memory (e.g., Phelps, 2004). Throughout development, the amygdala has also been shown to play a role in internalizing disorders such as depression and anxiety, as well as bipolar disorders, irritability, and other related mood disorders (e.g., Yang et al., 2010; Wiggins et al., 2016). One study found that lower SES (i.e., education) was associated with decreased amygdala volume and that, in turn, decreased amygdala volume was associated with internalizing symptoms (e.g., depression; Merz et al., 2017). However, the association with amygdala volume was only observed in adolescents and young adults (ages 13–21), and not younger children (3–12). Another paper (Sudimac et al., 2022), which investigated the effects of a 1-h walk in an urban environment vs. nature, found that amygdala activity decreased after walking in nature but not in an urban environment. These findings underscore the importance of understanding the effects of built environment on amygdala function and internalizing/externalizing behaviors.

The regions of the brain that are consistently active and interconnected at rest (e.g., medial prefrontal cortex (mfPFC), posterior cingulate cortex, angular gyrus) are referred to as the default mode network (DMN). The DMN has been shown to be related to numerous cognitive functions, such as social cognition, theory of mind, perspective taking, creating personal narratives, and daydreaming (Fox et al., 2005; Buckner et al., 2008). Moreover, the DMN, in relation to certain executive functions, is known to go through maturation (i.e., specialization) throughout adolescence (e.g., Sherman et al., 2014). Connections between the amygdala and regions in the DMN have also been implicated in the development and specificity of psychopathology. For example, increased connectivity between the amygdala and posterior cingulate gyrus, a central node of the DMN, has been shown to be related to higher rates of anxiety (Toazza et al., 2016). As such, amygdala-DMN connectivity makes for an intriguing target for investigation of the role of gray space exposure in the development of internalizing symptoms.

To our knowledge, limited studies have directly examined the effects of land characteristics on amygdala-DMN connectivity in adolescence (Kühn et al., 2017; Hackman et al., 2021; Rakesh et al., 2021). Studies have found adverse environmental experiences (e.g., low family income) associated with reduced volume within regions involved in the DMN (e.g., hippocampus, bilateral IPL, insula cortex, inferior frontal gyrus, right occipital, and mPFC; Noble et al., 2015). Rakesh et al. (2021) found that neighborhood disadvantage is associated with alterations in the DMN, and specific links have been reported between amygdala structure and forest coverage and green space in older adults (Kühn et al., 2017). Moreover, associations have been found between the amygdala and other aspects of lived environments (e.g., forest coverage, urban green space; Kühn et al., 2017). Forest coverage has been shown to be significantly associated with increased structural amygdala integrity in older adults (mean age = 70.1; Kühn et al., 2017).

Research has found that the presence of impervious surfaces (i.e., buildings, concrete, parking lots, etc.) is greater in neighborhoods with lower socioeconomic status and higher proportion of residents from minoritized backgrounds (Lu and Weng, 2007; Ogneva-Himmelberger et al., 2009). Urban settings with high levels of impervious surfaces (i.e., gray space), a lack of green space and vegetation, and greater density of buildings experience higher temperatures, greater heat stress, and greater thermal discomfort, especially in disadvantaged neighborhoods (i.e., areas with lower income and less access to resources for extreme weather; Harlan et al., 2006; Hajat and Kosatky, 2010).

This study aimed to expand previous literature on the link between socioeconomic status on amygdala-DMN connectivity by including built environment variables that extend beyond traditional operationalized variables of socioeconomic status. The Adolescent Brain Cognitive Development (ABCD) Study offers a unique opportunity to explore facets of the built environment among a large, diverse sample of the United States, with varying locations, socioeconomic statuses, and access to neighborhood resources. The goal of the present study was to broadly investigate the link between gray space on bilateral amygdala-DMN resting state functional connectivity (rs-fMRI) and associations with downstream behavioral outcomes including internalizing and externalizing symptoms. Particularly in a preliminary investigation, analyzing the DMN allows for a look into brain connectivity at a “baseline.” This type of imaging is particularly useful among youth, as performance on task-based imaging is less reliable and more variable in younger ages and across childhood. We did not have a directional hypothesis due to the lack of evidence from the literature supporting specific changes in connectivity in relation to gray space. As such, the current analyses present a preliminary investigation into the relationship between the presence of gray space, amygdala-DMN connectivity, and internalizing and externalizing symptoms.

## Materials and methods

All protocols were approved by the University of California, San Diego, and local site institutional review boards (IRBs) and informed consent and assent were obtained from caregivers and youth.

### Participants

The study used baseline data collected from the Adolescent Brain Cognitive Development (ABCD) study, a diverse, national, prospective, longitudinal study that recruited 11,878 youth and their caregivers (youth = 9–10 years old). Participants were excluded if youth are not fluent in English, had an MRI contraindication, major neurological disorder, gestational age less than 28 weeks or birth weight less than 1,200 grams, birth complications that resulted in hospitalization for more than 1-month, uncorrected vision, or current diagnosis of schizophrenia, autism spectrum disorder (moderate, severe), intellectual disability, or alcohol/substance use disorder.

At baseline, youth and one caregiver completed one to two in-person sessions, in which they completed a battery of assessments including mental and physical health (Barch et al., 2018), substance use (Lisdahl et al., 2018), peer, family, culture, and environment (Zucker et al., 2018), biological functioning (Uban et al., 2018), genetics (Iacono et al., 2018), and neuropsychological functioning, and MRI scans (Casey et al., 2018; Luciana et al., 2018). The current study utilized complete data from the demographic, residential history, and neuroimaging data (*n* = 10,144) (Fan et al., 2021). This project used ABCD 4.0 data release (2021) and was limited to a cross-sectional design using geo-coded primary address at baseline, as that is the most recently released data available.

### Measures

#### Geo-coded variables

**Primary residential address.** Primary residential addresses were collected from each participant’s caregiver at the baseline visit from 2016 to 2018 including questions such as “At what address does your child live?” Primary residential address was defined as the child spending at least 80% of their time at this current address (e.g., a child may have up to three current addresses that they live at one time). Each primary residential address was geocoded by the Data Analysis, Informatics, and Resource Center (DAIRC) of the ABCD Study in which they used the google map application programming interface (API) to generate latitude and longitude (Fan et al., 2021).^1^

**Defining gray space.** The Childhood Opportunity Index 2.0 (COI 2.0) was used to derive the gray space variable. The COI 2.0 is a measure that is comprised for all neighborhoods using census tracts in the United States and lends an overall index health and environment including a measure of access to green space (Acevedo-Garcia et al., 2020). More information on this particular measure can be found in Fan et al. (2021) and the COI 2.0 documentation (Acevedo-Garcia et al., 2014; Noelke et al., 2020). Based on the COI 2.0 measure, impervious surfaces are defined as the percentage of any area or land that is covered by impenetrable materials (e.g., brick, pavement, roads, rooftops; see Figure 1; Noelke et al., 2020) surrounding the place of residence for 30-meter pixels based on satellite imagery that was then aggregated to the census tract level (Dewitz and U.S. Geological Survey, 2021). Noelke et al. (2020) computed access to green spaces by standardizing imperviousness values to a z-score and multiplying by −1. For the present study, we utilized this variable before values are multiplied by −1 (i.e., the percentage of impervious surfaces).

**Neighborhood disadvantage.** Using geo-coded primary residential address at baseline, variables from the American Community Survey, a 5-year estimate between 2011 and 2015 were linked to individuals using the U.S. Census Tract which was used to calculate the area deprivation index (ADI) (Kind et al., 2014). The ADI is compiled of 17 sub-scores to capture neighborhood level deprivation; however, we selected five distinct variables often used in the measurement of disadvantage as previously cited in other work (Sampson et al., 1997; Morenoff et al., 2007; Diemer et al., 2013; Hackman et al., 2021) including median family income, percentage of residents with at least a high school diploma, unemployment rate, percentage of families living below the federal poverty level, and percentage of single-parent households.

Parental education was measured by self-reported highest level of educational attainment of both parents. This variable was a categorical variable with the following categories. (1) <HS Diploma, (2) HS Diploma/GED, (3) Some College, (4) Bachelor, and (5) Post Graduate Degree. Household income was measured by asking the parent their overall household income. The item asks “What is your total combined family income for the past 12 months? This should include income (before taxes and deductions) from all sources, wages, rent from properties, social security, disability and veteran’s benefits, unemployment benefits, workman.” Responses included 1 = Less than USD 50,000; 2 = USD 50,000–100,000; 3 = USD 100,000 +.

**Internalizing and externalizing symptoms.** The ABCD Study used a computerized Child Behavior Checklist, a self-report measure administered to the parents of youth investigating common internalizing, externalizing, and social behaviors among children and adolescents (Achenbach, 2009).

**Parental Monitoring and Warmth.** At baseline, items from the Child Report of Behavior Inventory (CRPBI, Schaefer, 1965; see also Barber, 1997) were used to assess youth’s perceptions of caregiver acceptance and were used as covariates in both models. Items were reported by youth to record on their caregiver participating in the study, for example biological mother, and a secondary caregiver chosen by the youth (e.g., father, grandparent). Responses were on a three-point scale reflecting parental warmth and acceptance (e.g., “makes me feel better after talking over my worries;” “smiles at me very often”).

The Parental Monitoring Scale (Karoly et al., 2015) assesses the extent to which the caregiver participating in the study has knowledge of their child’s whereabouts and the degree to which that intersects with shielding their youth from health-risk related behaviors. Details on the social-buffering effects and sociodevelopmental theory behind this measure are described in Zucker et al. (2018). The Parental Monitoring Scale has five questions on a scale ranging from never (1) to almost always (5).

#### Neuroimaging procedures

Imaging protocols for the ABCD study have been outlined in Casey et al. (2018). See Supplementary material for more information.

The amygdala was chosen *a priori* as a region of interest due to its association with the default mode network, disadvantaged neighborhoods and the environment (e.g., forest coverage, urban green space; Kühn et al., 2017; Rakesh et al., 2021), and relationship to internalizing symptoms and mood disorders (e.g., Yang et al., 2010; Wiggins et al., 2016). Pair-wise correlations were used for regions of interest (ROI) within defined parcellations, including the Default Mode Network (DMN) Gordon network (Gordon et al., 2016). The correlation values were transformed into Fisher-z-values and examined between the DMN and the subcortical ROIs, left and right amygdala.

### Analytic plan

To examine the relationship between the presence of gray space surrounding place of residence and amygdala-DMN connectivity, we specified two linear mixed effects models: One for left amygdala-DMN connectivity and one for right amygdala-DMN connectivity. In each of the linear mixed effects models, we specified one between-subjects factor (gray space) in relation to pre-calculated correlation values between the DMN and each respective amygdala seed. MRI manufacturer and relationship to family [i.e., siblings; in order to account for the nested structure of ABCD data (Saragosa-Harris et al., 2022)] were specified as random effects in the models. Age, sex, and five neighborhood disadvantage variables (median family income, percentage of residents with at least a high school diploma, unemployment rate, percentage of families living below the federal poverty level, percentage of single-parent households, parental monitoring, and parental warmth) derived from the ADI were included as covariates. Models were run with and without race and ethnicity as covariates due to neighborhood level variables potentially explaining more of the variance related to neighborhood-level variables of structural racism and racial segregation over and above individual level characteristics.

To examine the relationships between amygdala-DMN connectivity and internalizing and externalizing symptoms, we specified four correlation models, two for left amygdala-DMN connectivity and two for right-amygdala-DMN connectivity (with internalizing and externalizing symptoms separately for each).

## Results

The present sample comprised *n* = 9,091participants (female = 47.60%; male = 52.40%; white = 67.39%; Black = 13.63%; Asian = 2.25%; Other = 3.85%; Multi-racial = 12.24%; American Indian Alaska Native = 0.64%; Non-Hispanic = 81.09%; Hispanic = 18.91%) at baseline of the ABCD Study between 2016 and 2018. Table 1 summarizes additional participant demographics.

**TABLE 1:** Demographic characteristics.

	Total sample (*N* = 9,091)
**Sex, n (%)**	
Female	4,327 (47.60)
Male	4,764 (52.40)
Age, months [years], M (SD)	119.11 [9.92] (7.49)
**Household income, n (%)**	
<$50K	2,528 (27.80)
>$50 and <100K	2,607 (28.68)
>$100K	3,956 (43.52)
**Parent education, n (%)**	
<High school diploma	314 (3.45)
High school diploma/GED	706 (7.77)
Some college	2,331 (25.64)
Bachelor degree	2,428 (26.70)
Post graduate degree	3,312 (39.44)
**MRI manufacturer, n (%)**	
Phillips medical systems	1,011 (11.13)
GE medical systems	2,290 (25.19)
SIEMENS	5,790 (63.68)
Race, *n* (%)	(*n* = 9, 998)
White	6,126 (67.39)
Black	1,239 (13.63)
Asian	205 (2.25)
AIAN/NHPI	58 (0.64)
Other	350 (3.85)
Multi-racial	1,113 (12.24)
**Hispanic, n (%)**	
Yes	1719 (18.91)
No	7372 (81.09)
Impenetrable surface area (gray space), M (SD)	0.25 (0.92)
Federal poverty line,%	10.72 (11.25)
Unemployment,%	8.70 (5.62)
High school diploma,%	89.01 (10.81)
Median family income, *M* (SD)	$78,416.24 ($35,772.00)
Single parent household,%	17.22 (12.12)
Parental monitoring, *M* (SD)	4.39 (0.51)
Parental warmth, *M* (SD)	2.74 (0.29)

AIAN/NHPI, American Indian Alaska Native; Native Hawaiian Pacific Islander; M, mean; SD, standard deviation.

**Left amygdala-DMN.** More gray space, adjusting for age, sex, and neighborhood level variables, was significantly associated with increased left amygdala-DMN connectivity *(t* = 2.582, *p* = 0.001; Table 2, Model 1; see Supplementary Figure 1). When controlling for race, ethnicity, household income, highest level of parent education, parental monitoring, and parental warmth the model remained significant *(t* = 2.259, *p* = 0.02; Table 2, Model 2). For unadjusted analyses, see Supplementary Table 1. No significant relationships were observed from analyses examining the correlation between left amygdala-DMN connectivity and internalizing or externalizing symptoms.

**TABLE 2:** Model summary tables: left amygdala-DMN and gray space.

Model 1 estimates	Estimated	Standard error	Cohen’s *F*^2^
Intercept	−4.302e−02	2.051e−02	–
Gray space	2.871e−03	1.090e−03	8.53e−04
Age	−1.266e−04	1.173e−04	1.15e−04
Sex (Male)	7.385e−03	1.780e−03	1.78e−03
Single parent	6.429e−05	1.328e−04	2.76e−05
Federal poverty line	−2.770e−04	1.609e−04	3.47e−04
Unemployment	1.675e−04	2.288e−04	6.23e−05
High school diploma	−2.124e−04	1.203e−04	3.57e−04
Median family income	2.437e−08	3.700e−08	5.35e−05
Parental monitoring	6.597e−05	1.892e−03	1.21e−07
Parental warmth	1.366e−03	3.215e−03	1.79e−05
**Model variance explained**			
R^2^ fixed	0.003		
R^2^ fixed + R^2^ random	0.076		
Eta squared	0.000		
**Model 2 estimates**	**Estimated**	**Standard error**	**Cohen’s *F*^2^**
Intercept	−5.587e−02	2.519e−02	–
Gray Space	2.659e−03	1.177e−03	7.04e−04
Age	−7.711e−05	1.240e−05	4.27e−05
Sex (Male)	7.584e−03	1.886e−03	1.87e−03
Single parent	7.270e−05	1.482e−04	3.18e−05
Federal poverty line	−2.105e−04	1.755e−04	1.90e−04
Unemployment	2.401e−04	2.515e−04	1.18e−04
High school diploma	−9.842e−05	1.433e−04	5.99e−05
Median family income	3.310e−05	4.059e−08	9.15e05
Race (American Indian Alaska Native; Native Hawaiian Pacific Islander)			2.85e−04
*Asian*	−4.613e−03	1.325e−02	
*Black*	−5.674e−03	1.201e−02	
*Mixed*	−8.650e−03	1.193e−02	
*White*	−8.910e−03	1.171e−02	
*Other*	−1.176e−02	1.262e−02	
Hispanic	1.939e−03	2.831e−03	6.01e−05
Household income	5.429e−04	1.572e−03	1.65e−05
Parent education	5.688e−04	1.141e−03	3.38e−05
Parental monitoring	5.479e−04	2.038e−03	8.02e−05
Parental warmth	3.684e−04	3.418e−03	1.29e−06
**Model variance explained**			
R^2^ fixed	0.003		
R^2^ fixed + R^2^ random	0.089		
Eta squared	0.000		

**Right amygdala-DMN.** No significant relationship was observed from analyses examining the effect of gray space on right amygdala-DMN connectivity (*t* = 1.121, *p* = 0.263; see Supplementary Table 2) or from analyses examining the correlation between right amygdala-DMN connectivity and internalizing or externalizing symptoms.

## Discussion

The goal of the present study was to investigate the effect of gray space surrounding place of residence on amygdala-DMN connectivity. There is limited research investigating brain connectivity and gray space relationships as it relates to social drivers of health. Due to the novelty of the present study, and lack of extant literature on gray space and brain connectivity, we did not include directional hypotheses. While framed as a preliminary investigation, the use of the ABCD Study dataset allowed for increased power in our analyses. The present study found evidence of a relationship between gray space and left amygdala-DMN connectivity. We did not observe a significant relationship between gray space and right amygdala-DMN connectivity. This suggests the environment, such as gray space exposure, has a specific effect on left amygdala-DMN connectivity. Future research should further investigate the mechanism behind this specific effect. Additionally, there were no significant relationships observed between right or left amygdala connectivity and internalizing or externalizing symptoms. These findings underscore the importance of understanding how environmental factors (e.g., different land types) can contribute to brain function – in particular, affect and cognition.

Results from the present study suggest that presence of gray space may play a role in the development of emotional processing/regulation (via the amygdala). For example, increased exposure to gray space surrounding primary residential address resulting in increased left amygdala-DMN connectivity may represent dysregulation between these regions. These results relate to amygdala findings that aspects of the lived environment (e.g., forest coverage) can impact the brain (Kühn et al., 2017; Sudimac et al., 2022). Given that Sudimac et al. (2022) observed a decrease in amygdala activity after a 1-h walk in nature, but did not observe changes in amygdala activation as a result of a 1-h walk in an urban setting (i.e., no change in pre and post fMRI scans), we wanted to extend this area of investigation by examining a baseline (i.e., prolonged vs. a change in) measure of gray space exposure on amygdala connectivity. The present study found a detrimental effect of gray space on amygdala-DMN connectivity. These results may identify the potential interplay between the environment (i.e., both gray and green spaces) and neural development which may predispose individuals to future mental health problems. Future research may benefit from an investigation of type of land, including both gray and green spaces, and neural development and subsequent psychopathology.

Evidence on the directionality of amygdala-DMN connectivity on downstream emotion dysregulation is mixed. Some evidence has implicated reduced connectivity between regions of the DMN, including the posterior cingulate, and amygdala with downstream emotional dysregulation in patients with bipolar disorder and major depression (Veer et al., 2010; Townsend and Altshuler, 2012). Studies investigating amygdala resting state functional connectivity and affective and cognitive networks, including regions in the DMN (i.e., precuneus), found abnormal (i.e., both increased and decreased) connectivity in adolescents with major depressive disorder (Tang et al., 2018). Moreover, other research has also found increased amygdala-DMN connectivity in adolescents and young adults with anxiety (Toazza et al., 2016). Research has suggested that throughout development –, from adolescence into early adulthood – the ability to down-regulate (e.g., via the prefrontal cortex to the amygdala) improves and becomes more effective (e.g., Silvers et al., 2014). For example, Silvers et al. (2014) found that, compared to adolescents, young adults were more effectively (i.e., longer lasting) able to down-regulate their amygdalae to aversive stimuli, as mediated by the rostrolateral PFC. Taken together, these findings provide additional support for understanding how aberrant amygdala-DMN resting state-functional connectivity may contribute to downstream emotion dysregulation symptoms.

Evidence has suggested there is a connection between increased amygdala connectivity to regions implicated in emotion and sensory processing and increased internalizing symptoms (e.g., depression and anxiety) among youth. For example, one study found increased internalizing symptoms among young females was associated with increased amygdala RSFC and regions implicated in emotional processing (Padgaonkar et al., 2020). Qin et al. (2014) found in a sample of 8-year-olds increased anxiety symptoms were associated with increased RSFC of the left amygdala and regions related to sensory processing (Qin et al., 2014). However, the present study did not find a link between left amygdala-DMN connectivity and internalizing nor externalizing symptoms. While these analyses did not reveal a link, the neuroimaging findings may be more sensitive than behavioral measures of emotion regulation, thus may be picking up on an aspect of dysregulation that self-report measures are not. Given that these findings were among 9- and 10-year-olds, it’s important for future research to investigate across the developmental span to observe changes throughout adolescence, which is the peak onset of internalizing disorders. Importantly, given that changes in amygdala connectivity have been shown to be related to the development of psychopathology across the lifespan (e.g., Yang et al., 2010; Wiggins et al., 2016), further elucidating these relationships is imperative for both understanding mental health outcomes in children based on lived environment and making policy recommendations related to gray space and urban spaces.

According to Brown et al. (2019), scientists should include health inequities in the context of their discussion of their findings and dissemination of their results. Using the Coll et al. (1996) model of development, we controlled for variables of neighborhood level factors (median family income, percentage of residents with at least a high school diploma, unemployment rate, percentage of families living below the federal poverty level, and percentage of single-parent households) when looking at elements of the built and natural environment on brain development to incorporate an individual’s social position and environment in our model. In particular, the results from the present study, which showed that amygdala connectivity was not significantly linked with race or ethnicity, helps demonstrate how investigating other upstream social and structural drivers of health (e.g., gray space) is necessary for fully understanding brain-environment relationships as they relate to health inequity.

There is limited research in clinical science that addresses the direct impact of systemic and structural biases on health outcomes, particularly among youth. Moreover, there is little research investigating the mechanisms underlying health disparities that go beyond observed differences among individuals, particularly among minoritized backgrounds including ethnic, racial, and socio-economically disadvantaged backgrounds (Causadias et al., 2018). Historically, however, psychological science has investigated white backgrounds, and has seldom strayed beyond investigating observed differences, specifically neglecting the impact of how cultural and economic privilege shapes health outcomes that exist among white and more affluent individuals, suggesting a persistent bias in scientific research (Causadias et al., 2018) and perpetuation of scientific inequities. And, even in instances when race/ethnicity or socioeconomic status are taken into account (e.g., controlled for statistically), they are being used as proxy variables for these types of social drivers of health. Thus, psychological science should investigate the role of culture, systems, and environment among the development of all youth to shape theory, interventions, and public policy.

### Limitations

While our preliminary findings suggest that the presence of gray space in the youth’s lived environments is associated with increased left amygdala-DMN connectivity, there are several limitations that need to be discussed. First, it has been cited that the ABCD Study participants that are recommended for analysis (e.g., low noise data, low average motion, etc.) are more socioeconomically advantaged, less diverse, perform higher on neurocognitive assessments, and report better physical and mental health outcomes relative to those within the sample with higher noise and more restricted samples (Cosgrove et al., 2022). Furthermore, while a strength of the ABCD Study is that participants were recruited from schools which were selected based on characteristics such as race, ethnicity, SES, sex, and urbanicity (Garavan et al., 2018), participants were limited to 21 study sites which may contribute to the ecological validity of these findings and confounding factors. However, our current study attempts to consider historical and socio-cultural context and systemic biases as it relates to brain connectivity. Second, the ABCD Study data set does not include estimates for the presence of gray space across the lifetime, and this sample was limited to primary address at baseline. Thus, our sample included individuals that have lived at their current primary address for at least 80% of their time throughout the week, and individuals who may have stayed at this address for as little as a month up to 100% of their lifetime, limiting the generalizability of our findings. Once residential history has been collected in full for each participant, it will be imperative to assess lifetime exposure of gray space. Third, our findings are preliminary as they are cross-sectional and have relatively small effect sizes, thus we are limited in making a causal inference, particularly as this study occurred at the ages of 9–11, and brain development is an ongoing process that continues through young adulthood. Fourth, the dysregulation of the left amygdala-DMN connectivity could possibly be related to other factors (e.g., heat exposure, air pollutants, walkability, population density, etc.). Finally, given how the gray space variable is calculated in the ABCD Study, there are limitations on how much the data can be parsed. For example, the gray space variable in the present study is singular in nature, which, therefore, means it cannot be separated to represent different aspects of the environment (e.g., green and blue space). Studies with additional – and more detailed – measures of the built and natural environment will be able to better investigate these differences and their differential impacts. Future research is needed to determine the relationship between gray space exposure, green space, and other resilience factors, such as markers of distinct neighborhood advantage, intertwined with gray space exposure on brain development from early childhood into adolescence and young adulthood that is available within the ABCD study and other prospective studies.

## Conclusion

Our findings, presented as a preliminary investigation into the association between gray space and default mode network-amygdala connectivity, provide evidence that aspects of built environment are linked with brain connectivity in youth aged 9–11. Importantly, these findings add to a small – but growing – body of evidence underscoring the importance of public health relevance in investigating the impact of built environment on affective neural connectivity during development. Moreover, the present study, in line with previous literature, suggests that increased gray space is linked with dysregulation of the amygdala and default mode network. In addition to future work, which can build on and expand these findings, the present study can help to inform future policy on improving lived and built environments.

## Data availability statement

The original contributions presented in this study are included in this article/Supplementary material. The code and corresponding descriptions of the statistical procedures for the current project can be found at: https://github.com/harri585/grayspace/blob/9ef1eebb210350347541f235542f5592a208fb07/grayspace_github.R. Further inquiries can be directed to the corresponding author.

## Ethics statement

The studies involving humans were approved by the UC San Diego Institutional Review Board. The studies were conducted in accordance with the local legislation and institutional requirements. Written informed consent for participation in this study was provided by the participants’ legal guardians/next of kin.

## Author contributions

JH and ML: conceptualization and writing—original draft preparation. KL, CL, and CC-I: methodology and supervision, project administration, and funding acquisition. JH, ML, and CC-I: software and formal analysis. All authors contributed to review and editing and have read and agreed to the published version of the manuscript.

## Footnotes

1
https://developers.google.com/maps/documentation/elevation/overview
