# Supplementary material for: Gray space and default mode network-amygdala connectivity
Source: Front Hum Neurosci. 2023 Aug 30;17:1167786. doi: 10.3389/fnhum.2023.1167786 (PMC10498535; doi:10.3389/fnhum.2023.1167786)
Supplement: Supplementary file 1 [file Data_Sheet_1.pdf]

# Supplemental Materials

## Neuroimaging procedures

Youth completed the scan on a 3T scanner (e.g., General Electric, Phillips, or Siemens) using a 32-channel head coil with T1-weighted and T2-weighted structural scans (0.7mm isotropic). Youth underwent four 5-minute resting-state blood oxygenation level dependent (BOLD) scans while opening their eyes and fixating on a crosshair. Images were acquired in the axial view using an echo planar sequence. For more details on resting-state image parameters see (https://abcdstudy.org/images/Protocol_Imaging_Sequences.pdf; Casey et al., 2018).

Multi-Model Pressing Stress software package, a comprehensive piping analysis software, was used to normalize and time course detrend resting state functional connectivity data (Hagler et al., 2019). Motion, white matter, ventricles, and whole brain were signals of noninterest and were removed by generalized linear model regression (Casey et al., 2018). Additionally, images with excessive motion (e.g., displacement ≥ 5 contiguous frames, respiratory signals) were removed.

**Supplemental Figure 1**

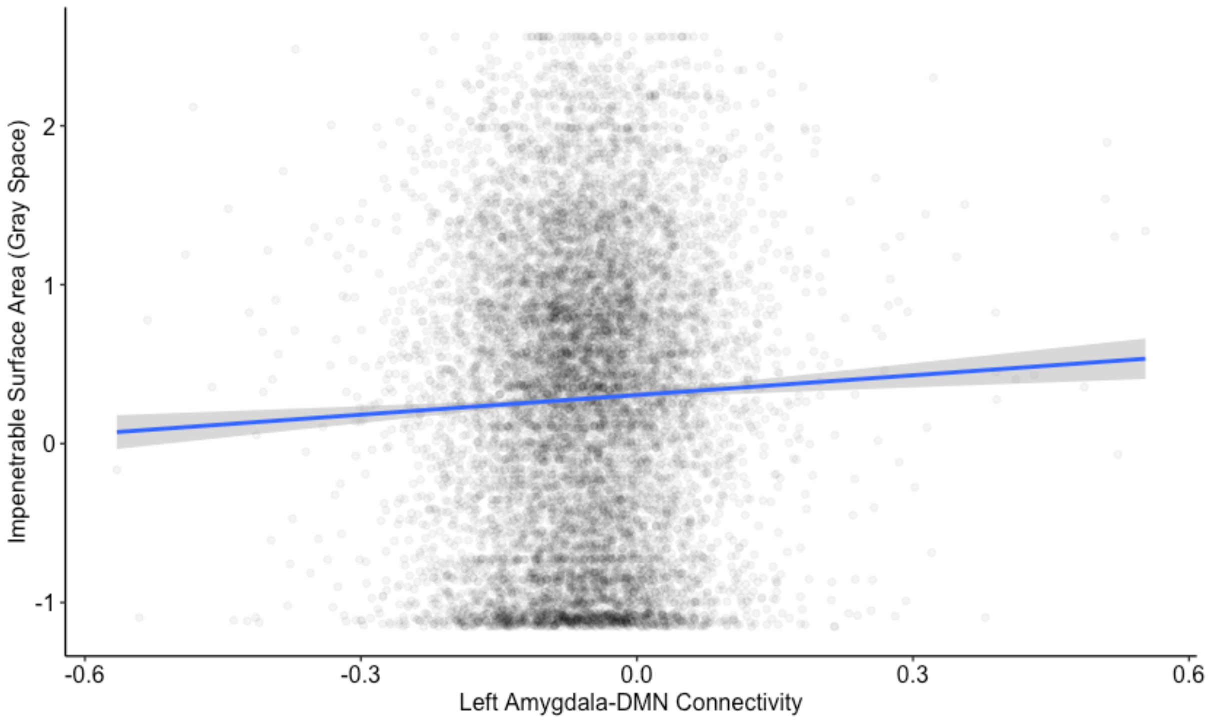


Supplemental Figure 1. Regression line (blue) and 95% confidence interval (gray) of the association between Left Amygdala-DMN Connectivity and Impenetrable Surface Area (Gray Space). The figure was set at alpha = 0.05.

**Supplemental Table 1 Model Summary Tables: Unadjusted Left Amygdala-DMN and Gray Space**

| Model 1 Estimates | Estimated | Standard Error | Cohen's $F^2$ |
|---|---|---|---|
| **Intercept** | **-6.006e-02** | **5.021e-03** | **-** |
| **Gray Space** | **2.802e03** | **9.534e-04** | **1.04e-03** |
| **Model Variance Explained** | | | |
| $R^2$ fixed | | 0.073 | |
| $R^2$ fixed + $R^2$ random | | 0.001 | |
| **Eta Squared** | | 0.001 | |

**Supplemental Table 2 Model Summary Tables: Right Amygdala-DMN and Gray Space**

| Model 1 Estimates | Estimated | Standard Error | Cohen's $F^2$ |
|---|---|---|---|
| **Intercept** | **1.681e-02** | **1.805e-02** | **-** |
| **Gray Space** | **1.216e-03** | **1.085e-03** | **1.78e-04** |
| **Age** | -2.433e-04 | 1.153e-04 | 4.42e-04 |
| **Sex (Male)** | **-8.056e-03** | **1.738e-03** | **2.19e-03** |
| **Single Parent** | -5.797e-04 | 1.324e-04 | 2.25e-03 |
| **Federal Poverty Line** | 1.126e-04 | 1.603e-04 | 5.75e-05 |
| **Unemployment** | -3.508e-04 | 2.281e-04 | 2.79e-04 |
| **High School Diploma** | 1.087e-04 | 1.197e-04 | 1.10e-04 |
| **Median Family Income** | 4.757e-08 | 3.700e-08 | 2.03e-04 |
| **Model Variance Explained** | | | |
| $R^2$ fixed | | 0.014 | |
| $R^2$ fixed + $R^2$ random | | 0.162 | |
| **Eta Squared** | | 0.000 | |

## References

Casey, B. J., Cannonier, T., Conley, M. I., Cohen, A. O., Barch, D. M., Heitzeg, M. M., Soules, M. E., Teslovich, T., Dellarco, D. V., Garavan, H., Orr, C. A., Wager, T. D., Banich, M. T., Speer, N. K., Sutherland, M. T., Riedel, M. C., Dick, A. S., Bjork, J. M., Thomas, K. M., Chaarani, B., … ABCD Imaging Acquisition Workgroup (2018). The Adolescent Brain Cognitive Development (ABCD) study: Imaging acquisition across 21 sites. Developmental cognitive neuroscience, 32, 43–54. https://doi.org/10.1016/j.dcn.2018.03.001

Hagler, D. J., Jr, Hatton, S., Cornejo, M. D., Makowski, C., Fair, D. A., Dick, A. S., Sutherland, M. T., Casey, B. J., Barch, D. M., Harms, M. P., Watts, R., Bjork, J. M., Garavan, H. P., Hilmer, L., Pung, C. J., Sicat, C. S., Kuperman, J., Bartsch, H., Xue, F., Heitzeg, M. M., … Dale, A. M. (2019). Image processing and analysis methods for the Adolescent Brain Cognitive Development Study. NeuroImage, 202, 116091. https://doi.org/10.1016/j.neuroimage.2019.116091
